# Vacuum Filtration-Coated Silver Electrodes Coupled with Stacked Conductive Multi-Walled Carbon Nanotubes/Mulberry Paper Sensing Layers for a Highly Sensitive and Wide-Range Flexible Pressure Sensor

**DOI:** 10.3390/mi15111306

**Published:** 2024-10-28

**Authors:** Guanhai Yan, Dongrui Dang, Sheng Chang, Xuefeng Zhang, Jinhua Zhang, Zhengdong Wang

**Affiliations:** 1 School of Mechanical and Electrical Engineering, Xi’an University of Architecture and Technology, Xi’an 710055, China; 2 Shaanxi Key Laboratory of Nano-Materials and Technology, Xi’an University of Architecture and Technology, Xi’an 710055, China

**Keywords:** flexible pressure sensor, conductive paper, multilayer structure, porous microstructures, resistive

## Abstract

Flexible pressure sensors based on paper have attracted considerable attention owing to their good performance, low cost, and environmental friendliness. However, effectively expanding the detection range of paper-based sensors with high sensitivities is still a challenge. Herein, we present a paper-based resistive pressure sensor with a sandwich structure consisting of two electrodes and three sensing layers. The silver nanowires were dispersed deposited on a filter paper substrate using the vacuum filtration coating method to prepare the electrode. And the sensing layer was fabricated by coating carbon nanotubes onto a mulberry paper substrate. Waterborne polyurethane was introduced in the process of preparing the sensing layers to enhance the strength of the interface between the carbon nanotubes and the mulberry paper substrate. Therefore, the designed sensor exhibits a good sensing performance by virtue of the rational structure design and proper material selection. Specifically, the rough surfaces of the sensing layers, porous conductive network of silver nanowires on the electrodes, and the multilayer stacked structure of the sensor collaboratively increase the change in the surface contact area under a pressure load, which improves the sensitivity and extends the sensing range simultaneously. Consequently, the designed sensor exhibits a high sensitivity (up to 6.26 kPa^−1^), wide measurement range (1000 kPa), low detection limit (~1 Pa), and excellent stability (1000 cycles). All these advantages guarantee that the sensor has potential for applications in smart wearable devices and the Internet of Things.

## 1. Introduction

With the development of information technology, the demand for high-performance flexible sensors for next-generation electronic devices, such as electronic skins [1,2,3,4], Internet of Things (IOT) devices [5,6], and soft robots [7,8], is rapidly increasing. Considerable progress has been made in the development of flexible pressure sensors based on different sensing mechanisms, materials, and structures [9,10,11,12,13]. However, to satisfy the demand for these emerging applications, flexible pressure sensors still face challenges in terms of having a high sensitivity, wide sensing range, fast response, and good stability [14]. According to the sensing mechanisms, flexible pressure sensors can be mainly divided into resistive [15,16,17], capacitive [18], piezoelectric [19], and triboelectric sensors [20,21,22]. Among them, resistive sensors, converting applied loadings into concomitant variations in resistance, have been widely investigated due to their simple signal conditioning and structural design [23,24,25,26,27]. However, traditional resistive flexible pressure sensors based on the piezoresistive effect of sensitive materials usually demonstrate a low sensitivity and narrow sensing range.

Recently, microstructures have been successfully introduced into the design of novel flexible resistive pressure sensors, which causes a significant increase in changes in the contact area once the sensor is compressed and results in prominent variations in resistance. As a result, this method is effective in improving the sensitivity and widening the sensing range [28,29,30,31,32,33]. However, most microstructures demand expensive and complicated manufacturing process, such as lithography and deposition, etc. [34]. In contrast, paper-based flexible pressure sensors have also garnered significant attention due to the low-cost and easily scalable process [35,36,37,38,39]. This can be attributed to the unique characteristics of paper. At first, paper possesses a highly porous cellulose fiber network, which provides sufficient deformation for the sensor under compression. In addition, paper demonstrates the advantages of a small thickness, good foldability/bendability, lightweight nature, renewability, and biocompatibility/biodegradability [40].

Several paper-based flexible pressure sensors have been reported in previous studies. A wearable pressure sensor based on MXene/Tissue Paper demonstrates an ultrahigh sensitivity of 509.5^−1^ in the range of 0.5–10 kPa and the maximum sensing range is only 100 kPa [41]. In contrast, a rigid–soft hybrid paper-based flexible pressure sensor is valid in an ultra-wide working range up to 1 MPa, but the maximum sensitivity is only 0.297 kPa^−1^ [42]. Consequently, it is still an urgent challenge to develop a high-sensitivity flexible pressure sensor with a wide sensing range. To overcome these disadvantages, mulberry paper has been employed in the design of paper-based flexible pressure sensors. The high content of holocellulose in mulberry paper endows it with a superior hydrophilicity [43,44] and makes it easier to integrate with aqueous conductive dispersion coatings. Moreover, the cellulose fibers in mulberry paper are stronger and longer than those in other types of paper [45], which effectively enhances its strength and greatly improves its foldability and bendability. With these good characteristics, mechanically robust mulberry paper has been used quite extensively in super capacitors [45], flexible electrodes [46], electromagnetic shielding [47], and flexible sensors [48,49]. The tactile sensor based on mulberry paper reported by Lee et al. shows a high sensitivity exceeding 1 kPa^−1^ in an ultra-wide range and proves the availability and efficiency of mulberry paper in the design of flexible pressure sensor; however, it still demands a complicated preparation process [49]. Additionally, the properties of the electrodes are crucial to the performance of the sensor too. Silver nanowires (AgNWs) are an excellent candidate for highly conductive electrodes with a hydrophilic substrate [50].

In this paper, a paper-based resistive pressure sensor has been presented that features a sandwich structure consisting of two AgNWs-covered filter paper (AgNWs/FP) electrodes and multilayer conductive mulberry paper (CMP) sensing layers. During the fabricating process of the CMP sensing layer, waterborne polyurethane (WPU) was introduced to improve the uniformity of the multi-walled carbon nanotubes’ (MWCNTs) dispersion and reinforce the interaction of the MWCNTs and the mulberry paper substrate [51]. Additionally, comparative studies have been conducted to investigate the effect of sensing layers prepared by drop coating and dip coating techniques as well as electrodes covered with AgNWs and MWCNTs on sensing performance, respectively. The results showed that both coating techniques were able to successfully form MWCNTs-conductive networks on surfaces of the mulberry paper substrate and had little effect on sensing performance. However, the electrodes coated with AgNWs demonstrated an absolute advantage over electrodes coated with MWCNTs in the sensing performance of the designed sensors. In conclusion, the rough surfaces of the sensing layers, porous conductive network of silver nanowires on the electrodes, and the multilayer stacked structure of the designed sensor synergistically provide the sensor with an excellent performance. Specifically, the designed sensor achieved a limit of detection of about 1 Pa and high sensitivities of 6.26, 4.69, and 1.707 kPa^−1^ in the pressure regions of 0–50 kPa, 50–500 kPa, and 500–1000 kPa, respectively. Thanks to the proper material selection and the rational structure design of the sensor, the high sensitivity and wide sensing range could be implemented simultaneously. Moreover, the sensor exhibited a stable response to 1000 cyclic loads and demonstrated excellent durability. Finally, based on the excellent performance of the prepared sensor, we successfully demonstrated the usability of the sensor in monitoring human activities, indicating that the sensor has significant application prospects in field of wearable devices.

## 2. Materials and Methods

### 2.1. Materials

Mulberry paper was purchased from Sufangzhai Co., Ltd., Xuancheng, China. Filter paper was purchased from Shanghai Xinya Purification Equipment Co., Ltd., Shanghai, China. MWCNTs (outside diameters of 30–80 nm, lengths > 10 µm, and purity > 98%) were purchased from Chengdu Organic Chemistry Co., Ltd. Chengdu, China. WPU, purchased from Shenzhen Jitian Chemical Co., Ltd., Shenzhen, Chian, has a solid content of 32 ± 5% and viscosity < 300 mPa·s. N,N-Dimethylformamide (DMF) was purchased from Chinasun Specialty Products Co., Ltd., Suzhou, China. AgNWs (diameters of 90 nm) was purchased from XFNANO Inc, Nanjing, China. Conductive silver paste was purchased from Hong Kong mechanic Co, Hongkong, China. All these materials and reagents were used as received.

### 2.2. Preparation of CMP Sensing Layers

**Figure 1:**
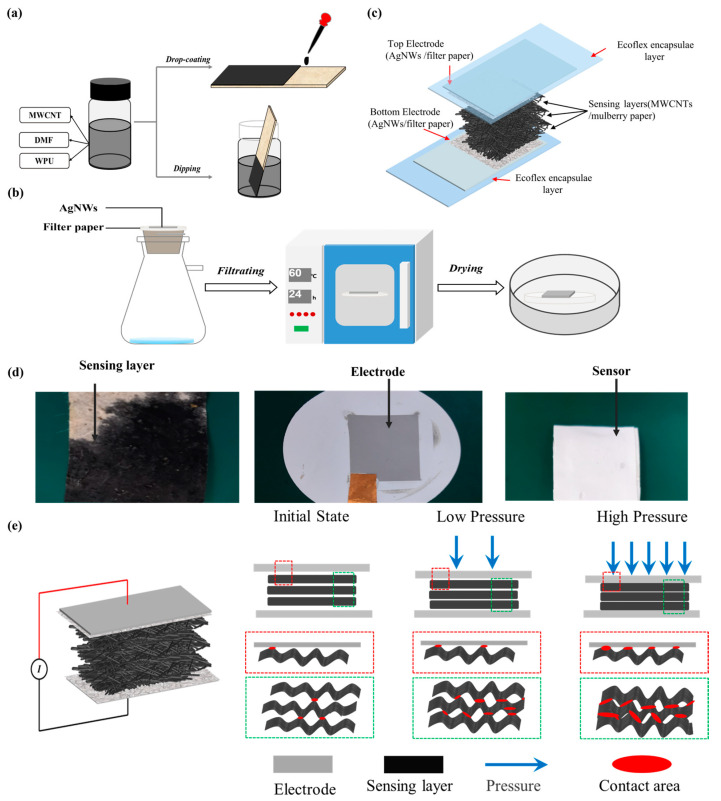
Schematic illustration of the fabrication process and sensing mechanism of the pressure sensor. (**a**) Preparation process of a CMP sensing layer. (**b**) Preparation process of a AgNWs/FP electrode. (**c**) Schematic illustration of the proposed all-paper-based flexible pressure sensor. (**d**) Digital images of a CMP sensing layer, a AgNWs/FP electrode, and a sensor, respectively. (**e**) Sensing mechanism of the proposed all-paper-based pressure sensor.

A certain amount of MWCNTs was uniformly dispersed into 3 mL of DMF by ultrasonic dispersion for 30 min under 40 °C. After 0.3 g WPU was added into the prepared MWCNTs/DMF dispersion, and vigorous stirring was conducted for 4 h. After that, another 1 h ultrasonic dispersion was performed to obtain the final conductive MWCNTs ink. Then, the mulberry paper coated with conductive MWCNTs film can be obtained by dip coating or drop coating method, as shown in Figure 1a. Finally, the CMP sensing layer was formed by annealing the mulberry paper coated with MWCNTs ink in a vacuum oven (70 °C for 30 min).

### 2.3. Preparation of AgNWs/FP Electrode

The AgNWs/FP electrode was prepared using the vacuum filtration coating method. In brief, an aqueous solution of silver nanowires (≈1 mg/mL) was prepared via an ultrasonic process for 5 min. Then, the conductive AgNWs film was transferred onto the surface of a piece of filter paper by vacuum filtration for 30 min. After that, the filter paper covered with a layer of conductive AgNWs film was put into a vacuum oven to be annealed at 60 °C for 24 h to form the final electrode. The MWCNTs/FP electrodes were prepared through an identical process and under the same conditions with different contents of MWCNTs. The process of preparing the AgNWs/FP electrode is illustrated in Figure 1b.

### 2.4. Fabrication of Pressure Sensor

The prepared CMP sensing layers and AgNWs/FP electrodes were cut into square pieces of 1 cm × 1 cm and 1.1 cm × 1.1 cm, respectively. Multiple CMP sensing layers were stacked layer by layer and sandwiched between two AgNWs/FP electrodes, as shown in Figure 1c. To facilitate the measurement of sensors, silver paste was applied to both electrodes to connect them with the copper foils. After being sealed withdouble-sided tape (Deli group) around the edges of electrodes, a paper-based flexible pressure sensor was obtained.

### 2.5. Characterization

Surface and cross-sectional morphology analyses of mulberry paper, filter paper, and CMP sensing layer were performed on a scanning electron microscope (SEM, GeminiSEM500, Carl Zeiss Microscopy GmbH, Oberkochen, Germany). Raman analysis of the mulberry paper and CMP sensing layer was performed on a Raman Spectrometer (LabRAM HR Evolution, HORIBA France SAS, Montpellier, France) with a 532 nm laser source. A measurement system was constructed to investigate the sensing performance of the fabricated sensor, as shown in Figure S1. The system included a universal testing machine (ZQ-990/LB, Zhiqu Precision Instruments Co., Ltd., Dongguan, China), a digital multimeter (Keysight 34465A, Keysight Technologies, Inc. Santa Rosa, USA), a computer, and the LABARD control software V 1.0 (Zhiqu Precision Instruments Co., Ltd., Dongguan, China). The constant driving voltage for the performance test of the designed sensor is 1 V.

## 3. Results and Discussion

### 3.1. Design Principle and Sensing Mechanism of the Pressure Sensor

According to the process used to fabricate the CMP sensing layer described in the last section, the properties of the conductive MWCNTs ink dictate the performances of the CMP sensing layers and its specific preparation process is shown in Figure S2. During the preparation process, WPU was innovatively introduced as a dispersant to manipulate the conductivity and coating ability of the ink. This is due to the fact that WPU, as a super dispersant containing hydrophilic groups, can increase the number of charges on the surfaces of MWCNTs and strengthen the electrostatic repulsion among MWCNTs, which improves the uniformity and stability of the MWCNTs/DMF dispersion. At the same time, the good coating ability of WPU enables MWCNTs to be tightly and uniformly wrapped onto surfaces of the fibers in mulberry paper, thus improving the mechanical properties (flexibility, toughness and robustness) of the CMP sensing layer. The obtained CMP sensing layer shows a highly uniform distribution of MWCNTs without delamination and exfoliation, as shown in Figure S3. As demonstrated in Figure 1b, both drop coating and dip coating are available for the preparation of the CMP sensing layers. When drop coating is adopted, conductive MWCNTs ink should be applied on both sides of the mulberry paper to assure the good conductivity of the sensing layer. The fabricated sensing layer, electrode, and pressure sensor are shown in Figure 1d, respectively. The designed sensor mainly depends on the variations in contact resistances between the different layers to realize the pressure measurement (Figure 1e). Few initial interactions between the CMP sensing layers and electrodes and a high resistance value will occur as soon as the sensor encapsulation has been completed. And each layer of the sensor will deform accordingly when the sensor is under an applied pressure. Therefore, the contact area and electron transport paths between sensing layers and electrodes as well as among different CMP sensing layers increase gradually, which results in a decrease in resistance. As a result, a change in pressure can be inferred from the varying resistance. Multilayer stacked sensing layers not only provide a greater variation in contact area but also distribute the applied pressure among different layers; then, a high sensitivity and a wide sensing range can be achieved simultaneously. At the same time, microstructures on the surfaces of electrodes and CMP sensing layers as well as the flexible porous structures distributed in the CMP sensing layers make surfaces coarser and interlock each layer to hinder delamination during operation and to improve the robustness of the device.

According to the working mechanism described above, the content of MWCNTs dominates the resistivity of the CMP sensing layers and then determines the performance of the designed sensors. The resistivity of a CMP layer decreases from ~2.9 kΩm to ~0.67 kΩm as the content of MWCNTs in the conductive ink increases from 6.25 wt% to 9.09 wt% (Figure S4). It is clear that a high resistance value increases the difficulty of signal conditioning but a small resistance narrows the sensing range. Finally, the conductive ink with a MWCNTs content of 7.69 wt% is optimized for preparing the CMP sensing layers.

### 3.2. Characterization of the Pressure Sensor

Mulberry paper, made of long and thick fibers, possesses a series of rough microstructures and a coarse surface morphology. As a result, the thickness of mulberry paper (325 μm) is much larger than that of copy paper (98 μm), as shown in Figure S5. Essentially, the porous structure of mulberry paper comes out of the thicker and looser fibrous structure of the mulberry paper. During the drying process after drop coating or dip coating, dehydration of the mulberry paper fibers will initiate in the secondary crinkled microstructures of the CMP sensing layers. These two microstructures synergistically endow the designed sensor with unique sensitive properties, meaning that a high sensitivity and wide sensing range can be achieved simultaneously.

**Figure 2:**
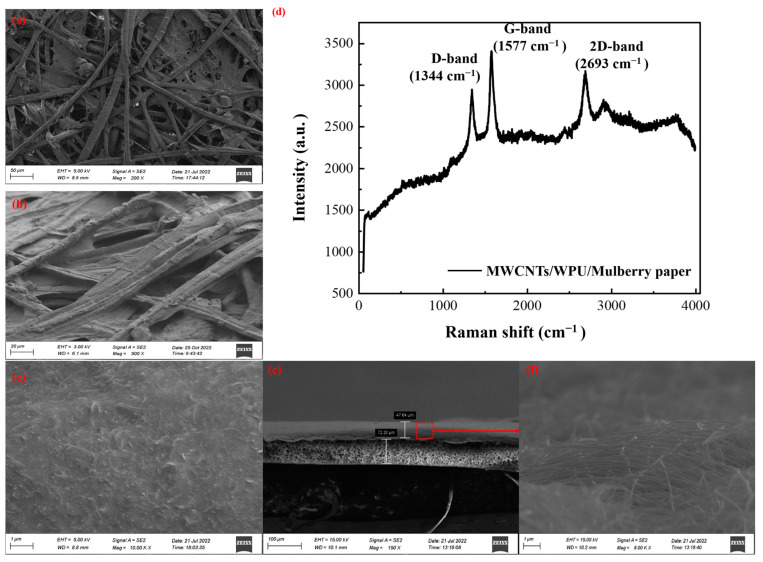
Characterization of all-paper-based flexible pressure sensor. (**a**) SEM image of pristine mulberry paper. (**b**) Surface SEM image of a CMP sensing layer prepared by dip coating process. (**c**) An enlarged view of a CMP sensing layer SEM image. (**d**) Raman spectra of a CMP sensing layer. Characteristic peaks of MWCNTs, WPU, and mulberry paper are clearly depicted. (**e**) Cross-sectional SEM image of a AgNWs/FP electrode. (**f**) Enlarged view of figure (**e**).

Both drop coating and dip coating processes have been employed to prepare the CMP sensing layers. The SEM image of pristine mulberry paper is shown in Figure 2a. SEM images of the CMP sensing layers prepared by the dip coating processes are shown in Figure 2b,c. In addition, SEM images of the CMP sensing layers prepared by drop coating processes are shown in Figure S6. From these figures we can see that both coating processes can successfully distribute MWCNTs on the surfaces of mulberry papers without changing the intrinsic fiber structures. Interestingly, the MWCNTs are distributed both on the fibers and in the spaces among the fibers to constitute a porous and stacked conductive structure on the surfaces of the CMP sensing layers. As the number of dropping or dipping process increases, there is a cumulative increase in the content of MWCNTs in the CMP sensing layers (Figure S7). At the same time, the resistivity of the CMP sensing layer decreases with the increasing number of dip coatings, as shown in Figure S8. There is a rapid drop in the resistivity of the CMP sensing layer after four times of dip coating. As discussed in the section on the sensing mechanism, sensing layers with extreme low resistivities will reduce the sensitivity, and so the optimum number of dip coating was determined to be three. Conductive paths among MWCNTs were successfully established using the mulberry paper completely coated with MWCNTs, which suggests that both the drop coating and dip coating are effective preparation processes for CMP sensing layer. This can be confirmed by the results of the Raman spectra of materials too. Three characteristic peaks, D-(1344 cm^−1^), G-(1577 cm^−1^), and 2D-(2693 cm^−1^), are clearly shown in the Raman spectra of the CMP sensing layer (Figure 2d). By comparing it with the Raman spectra of MWCNTs, WPU, and pristine mulberry paper (Figure S9), we find out that both dip coating and drop coating can form conductive MWCNTs networks on the mulberry paper without any evidential modifications to the structures of MWCNTs and mulberry paper.

The electrodes in the pressure sensor employ a piece of filter paper as a substrate and AgNWs as conductive materials. Vacuum filtration coating was conducted to transfer the conductive AgNWs film onto the filter paper substrate. SEM images of a prepared electrode show that a conductive AgNWs film with uniform thickness was evenly formed on the surface of the filter paper (Figure 2e,f). As illustrated in the figures, silver nanowires demonstrate a random interlacing distribution with a certain number of gaps among them forming a porous conductive film. Additionally, the strong binding interaction of the AgNWs and the paper substrate can also seize the conductive nanowires during bending. Consequently, the properties of both the structure and the material facilitate the formation of robust conductive paths, which makes the AgNWs/FP electrode present excellent performance. To illustrate the reliability of the prepared electrodes in a bending state, the electrodes were fixed on curved surfaces with different diameters and the resistances were recorded and analyzed (Figure S10). The AgNWs/FP electrode presents a small and stable resistance value even after being bent, demonstrating its high reliability. For comparison, the electrodes with conductive MWCNTs films were also prepared by the same process. However, the MWCNTs/FP electrodes exhibit a large resistance value and violent fluctuation, indicating their poor performance as electrodes. To some extent, this poor performance can be ascribed to the thicker and more heterogeneous conductive film composed of the MWCNTs, as shown in Figure S11. Based on these results, the pressure sensor with AgNWs/FP electrode was adopted for the subsequent experiments because of its good reliability and high conductivity.

### 3.3. Pressure Sensing Performances of the CMP Sensors

**Figure 3:**
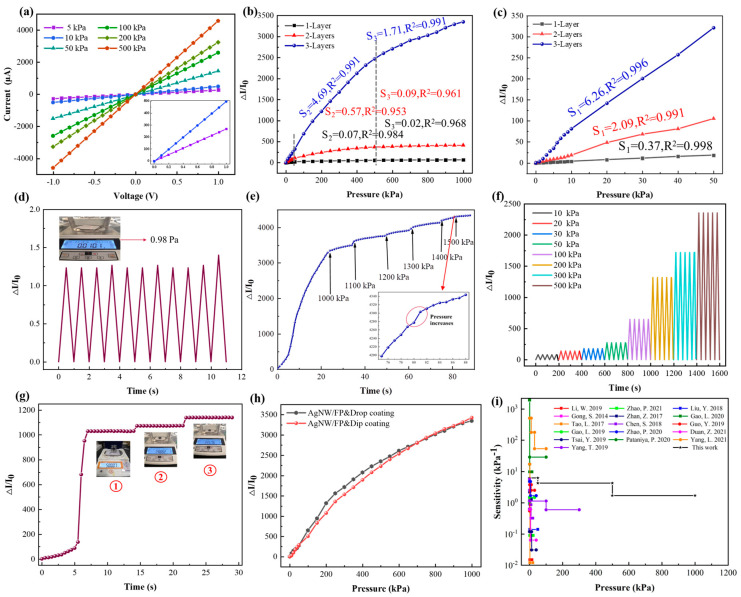
Pressure sensing performance of the CMP sensors. (**a**) Current-voltage curves of the three-layer CMP pressure sensor under different pressures varying from 5 kPa to 500 kPa. (**b**) Relative current changes in pressure sensors with one, two, and three CMP sensing layers in the pressure range of 1–1000 kPa. (**c**) Relative current changes in pressure sensors with one, two, and three CMP sensing layers in the pressure range of 1–50 kPa. (**d**) Dynamic response of the sensor under repeated pressure of 0.98 Pa for 11 cycles. (**e**) Current change in the sensor under a pressure over 1000 kPa. (**f**) Dynamic response of the pressure sensor under different pressures within 10–500 kPa. (**g**) Small pressure detection under high pressure. Three weights of 500 g, 1.48 g, and 1.45 g were placed on top surface of the sensor one after another, denoted in three stages denoted as ➀, ➁, and ➂. (**h**) Performance comparison of sensors prepared by drop-coating and dip-coating process. (**i**) Comparisons of sensitivity and sensing range with previously reported paper-based pressure sensors [15,41,54,55,56,57,58,59,60,61,62,63,64,65,66,67].

The sensing performance of the designed CMP sensor was examined with an experimental apparatus (shown in Figure S1). The current–voltage (I–V) curves of the designed sensor with three CMP sensing layers under various pressures are depicted in Figure 3a. From the shape of all the I–V curves we can find that the designed sensor exhibits a decrease in resistance with increasing pressure. The relative changes in the current of the sensors with a different number of CMP sensing layers are shown in Figure 3b,c. As shown in these Figures, all the sensors demonstrate an increase in current but the degree of change decreases with the increasing pressure. The sensitivity S is defined to quantitatively characterize the sensing performance of the designed sensor. Specifically, S = (∆I/I_0_)/P, where ∆I is the relative current change, I_0_ is the initial current, and P is the applied pressure. The whole range of applied pressures is divided into three sections to satisfy the requirement that the coefficient of determination (R^2^) of each section is higher than 0.95 [35]. According to the definition of sensitivity, the calculated sensitivities of sensors based on one-layer, two-layer, and three-layer CMP structures in the pressure range of 1–50 kPa are 0.37 (R^2^ = 0.998), 2.097 (R^2^ = 0.991), and 6.26 kPa^−1^ (R^2^ = 0.996), respectively. Similarly, the corresponding sensitivities in the range of 50–500 kPa are 0.077 (R^2^ = 0.984), 0.575 (R^2^ = 0.953), and 4.69 kPa^−1^ (R^2^ = 0.991), and are 0.018 (R^2^ = 0.968), 0.089 (R^2^ = 0.961), and 1.707 kPa^−1^ (R^2^ = 0.991) in the range of 500–1000 kPa, respectively. The most probable reason for the different sensitivities under various pressures is stated below. In a low-pressure regime, the microstructures in the CMP sensing layers and AgNWs/FP electrode can effectively concentrate loads and form more conductive paths, leading to a sharp decrease in resistance with increasing pressure. Nevertheless, the deformation and contact area of sensing layers and electrodes gradually reach saturation as pressure further increases, which leads to a decreased sensitivity. Compared with the sensors that consist of one or two CMP sensing layers, the sensor with three CMP sensing layers exhibits a higher sensitivity in a wide sensing range. This may be explained by the fact that more abundant variations in the contact area will be introduced when the sensor comprises more sensing layers. An electromechanical model was established to elaborate the principle of the change in the resistance of sensors with a different number of CMP sensing layers (Figure S12). It is clear that the designed sensor exhibits similar characteristics as the sensitivity is significantly improved with the increasing number of CMP sensing layers as previously reported [52]. In addition, a sensor with four CMP sensing layers was also prepared and investigated. However, the high initial resistance (>3.2 MΩ) accompanied by significant fluctuations makes it difficult to implement a practical measurement, as discussed in previous research [53]. Hence, sensors with three CMP sensing layers were used to conduct the subsequent measurements. Besides the sensitivity and sensing range, the limit of detection (LoD) is another crucial performance parameter for flexible pressure sensors. Thanks to the porous microstructures of both the CMP sensing layers and electrodes as well as the multilayer stacked structure of the sensor, the LoD of the designed sensor can be as low as 0.98 Pa (Figure 3d).

Additionally, the measurement capability of the presented sensor in a high pressure range (>1000 kPa) is illustrated in Figure 3e. As shown in this Figure, the relative change in current is tiny when the pressure is greater than 1000 kPa. And almost no more changes in current can be observed when pressure is greater than 1500 kPa, indicating that full contact has been nearly established in the sensor and it has reached the upper limit of measurement. Figure 3f shows that the sensor can accurately discriminate a series of repeated pressures varying from 10 kPa to 500 kPa. The relative variation in current steadily increases with increasing pressure and shows no significant difference for identical pressures. Figure 3g confirms that the designed sensor possesses high resolution under high pressure, which can be attributed to the high sensitivity and wide sensing range.

The stability and the durability of the presented sensor were evaluated by two experiments with repeated loading. Two pressures of 100 kPa and 500 kPa were intermittently loaded on the sensor for 1000 cycles respectively. The output currents of the sensor under corresponding pressure are presented in Figure S13. From this Figure we can see that designed sensor presents a highly stable change in current during the experiment, indicating its immense superiority and potential in long-term operations. The hysteresis characteristics of the presented sensor are shown in Figure S14. The presented sensor exhibits a hysteresis error of about 2.2% during the dynamic loading and unloading process from 0 to 500 kPa, which indicates its reasonable stability. Furthermore, to verify the reproducibility of the presented method, comparative investigations into the performances of three different sensors fabricated using identical process were conducted. The experimental results are shown in Figure S15. Despite slight differences in pressure sensing performances, all the sensors exhibit high sensitivities in a wide sensing range. In summary, the high porosity and good robustness of the CMP sensing layers and the excellent conductivity of the AgNWs/FP electrodes provide the sensor with a good reliability and reproducibility.

As mentioned in Section 3.2, both drop coating and dip coating processes are available to successfully coat the conductive MWCNTs onto the surfaces of fibers in mulberry paper. Furthermore, the sensing performance of sensors consisting of different CMP sensing layers were investigated and compared to study the effect of preparation process of CMP sensing layers on the performance of sensors. The result clearly shows that the preparation process used for the CMP sensing layers has little effect on their sensing performance, as illustrated in Figure 3h. The most significant factor is the porous structure and surface property of mulberry paper as well as the nature of the MWCNTs facilitate the uniform and consistent distribution of MWCNTs and stable interfacial bonding between the MWCNTs and mulberry paper.

Despite its low conductivity, the MWCNTs/FP electrode was also adopted to encapsulate MWCNTs/FP pressure sensors for further investigations. The sensing performances of the MWCNTs/FP sensors are illustrated in Figure S16. By comparing Figure S16 and Figure 3h, we can clearly see the difference in sensing performance between the two types of sensors. Sensors with MWCNTs/FP electrodes demonstrate a much lower sensitivity than that of sensors with a AgNWs/FP electrode. This may be attributed to the thicker and more compact conductive MWCNTs film deposited on the surface of the filter paper during the vacuum suction process. This dense and compact conductive film on the electrode only provides a limited variation in contact area and resistance when the sensor is loaded. As a result, the measured sensitivity is lower than that of sensors with AgNWs/FP electrodes. Henceforth, sensors with AgNWs/FP electrodes are employed for the subsequent experiments.

A comparison between the sensing performances of the presented sensor and other previously reported paper-based sensors is presented in Figure 3i [54,55,56,57,58,59,60,61,62,63,64,65,66,67] (the details are listed in Table S2). By comparing with relevant results, it can be found that the sensor presented in this paper demonstrates advantage in sensitivity over most sensors previously reported. Although a few sensors demonstrated higher sensitivities, they were usually constrained to narrower sensing ranges, such as 15 kPa [61], 20 kPa [62], and 100 kPa [41,67], respectively. In summary, the presented sensor consisting of multilayered CMP sensing layers and AgNWs/FP electrodes demonstrates a great potential for practical measurement because of its high sensitivity in an extra wide sensing range. All these results confirm that a CMP sensing layer with good conductivity, high porosity, and rough surfaces as well as a AgNWs/FP electrode with excellent conductivity are conductive to improve the sensing performance of the designed sensor.

### 3.4. Some Practical Applications of the CMP Sensor

**Figure 4:**
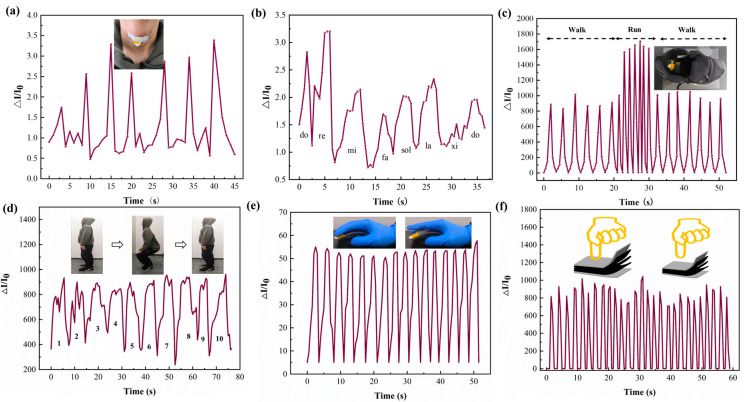
Some practical application examples of the presented CMP pressure sensors. (**a**) Measurement of tremor signals from the throat of a volunteer. (**b**) The relative change in current of the sensor caused by muscular activity of speaking. (**c**) Plantar pressure measurement achieved by a sensor fixed on an insole. (**d**) Plantar pressure signals measurement during a series of continuous squatting activities. (**e**) Real-time response of continuous mouse click. (**f**) Real-time response of continuous pressing on the top surface of a sensor.

The prepared sensor consisting of CMP sensing layers and AgNWs/FP electrodes exhibits a high sensitivity, wide sensing range, and long-term stability simultaneously. Some measurements were conducted to illustrate the availability of the designed sensor as wearable devices and human–machine interface. Firstly, the prepared sensor was fastened onto the surface of the throat of a volunteer to monitor the tremor signal caused by muscle activity (Figure 4a). A cough causes the sensor to produce a corresponding current change, and the amplitude of the current change reflects the contraction and relaxation activities of the muscle. And afterwards, the sensor was used to monitor the activity of pronunciation. The current curves corresponding to different musical notes of “do, re, mi, fa, sol, la, xi, do” are shown in Figure 4b. The results of these two experiments demonstrate that the sensor is capable of detecting tiny pressure variations and has potential for applications in speech recognition in the relevant fields.

To explore the applications of the presented sensor in a medium-high pressure range, the sensor was stuck on an insole to monitor the plantar pressure induced by different activities. Figure 4c illustrates the real time response of the sensor over a period of time with walking and running activities. It is clear that the corresponding signals represent the different activities clearly. At the same time, the sensor can capture the variation in plantar pressure during continuous squat training, as shown in Figure 4d. All of these results demonstrate that the presented sensor is able to detect plantar pressures with a high resolution and fast response time. The usability of CMP sensors in tactile perception was also studied in this paper. Finger touching with different pressures and frequencies can be detected by the sensor not only on a flat but also on a curved surface in real time (Figure 4e,f). The sensor was also used to control the brightness of three LEDs connected in parallel (Figure S17), indicating its robust and swift response to the applied pressure.

## 4. Conclusions

In this paper, we presented a paper-based resistive pressure sensor by stacking three CMP sensing layers between two AgNWs/FP electrodes. The unique rough surface and porous network structure of the CMP sensing layers, the excellent conductivity of the AgNWs/FP electrodes, and the stacked structure of the AgNWs/FP electrodes and CMP sensing layers synergistically create more significant changes in contact area, which contribute to considerable resistance change and improves the sensitivity effectively. Meanwhile, the adopted stacked multilayer structure can distribute the stress throughout each layer and widen the sensing range efficiently. As a consequence, the sensor performs with a high sensitivity of 6.26, 4.69, and 1.707 kPa^−1^ in the range of 1~50 kPa, 50~500 kPa, and 500~1000 kPa, respectively. At the same time, the high repeatability, good robustness, and long-term stability of the sensor were all verified by a series of experiments. With its significant advantages, the designed sensor was used as a wearable device for detecting human physiological signals. The presented sensor with high performances and low-cost, scalable preparation process will have a significant potential value for use in flexible electronic devices in the future.

## Data Availability

All relevant data are within the manuscript and its Supplementary Materials.

## Supplementary Materials

The following supporting information can be downloaded at: https://www.mdpi.com/article/10.3390/mi15111306/s1, Figure S1: Schematic of the experimental device that was used for investigation of the sensing performances. Figure S2: Preparation process of conductive MWCNTs ink. Figure S3: Pictures of CMP sensing layers. Figure S4: The resistivity of CMP varied with the content of MWCNTs in the conductive ink. Figure S5: Cross-sectional SEM images and thickness of mulberry paper and commercial A4-printing paper. Figure S6: SEM images of CMP sensing layer prepared by drop coating. Figure S7: SEM image of CMP prepared by dip-coating process with different number of times. Figure S8: Resistivity of CMP changes with number of dip-coating. Figure S9: Raman spectra of MWCNTs, WPU and pristine mulberry paper respectively. Figure S10: Reliability test for electrodes on curved surfaces. Figure S11: SEM images of MWCNT/FP electrode. Figure S12: Schematic diagram of the equivalent circuit of sensors with one, two, and three MCMP sensing layers. Figure S13: Repeatability test of the presented pressure sensor for repeated loading of 1000 cycles. Figure S14: Hysteresis test of the presented sensor. Figure S15: Consistency test of the designed sensor. Figure S16: Relative change in current of the three layers CMP sensor with MWCNT/FP electrodes. Figure S17: A designed sensor is used to control the brightness of the LEDs. Table S1: Composition of sensing layers with different content of MWCNTs; Table S2: Comparison of pressure-sensing performances with respect to sensitivity and sensing range among reported paper-based tactile sensors.
