# Supplementary material for: Vacuum Filtration-Coated Silver Electrodes Coupled with Stacked Conductive Multi-Walled Carbon Nanotubes/Mulberry Paper Sensing Layers for a Highly Sensitive and Wide-Range Flexible Pressure Sensor
Source: Micromachines (Basel). 2024 Oct 28;15(11):1306. doi: 10.3390/mi15111306 (PMC11596462; doi:10.3390/mi15111306)
Supplement: Supplementary file 1 [file micromachines-15-01306-s001.zip › micromachines-3252168-supplementary.pdf]

Supplementary Materials

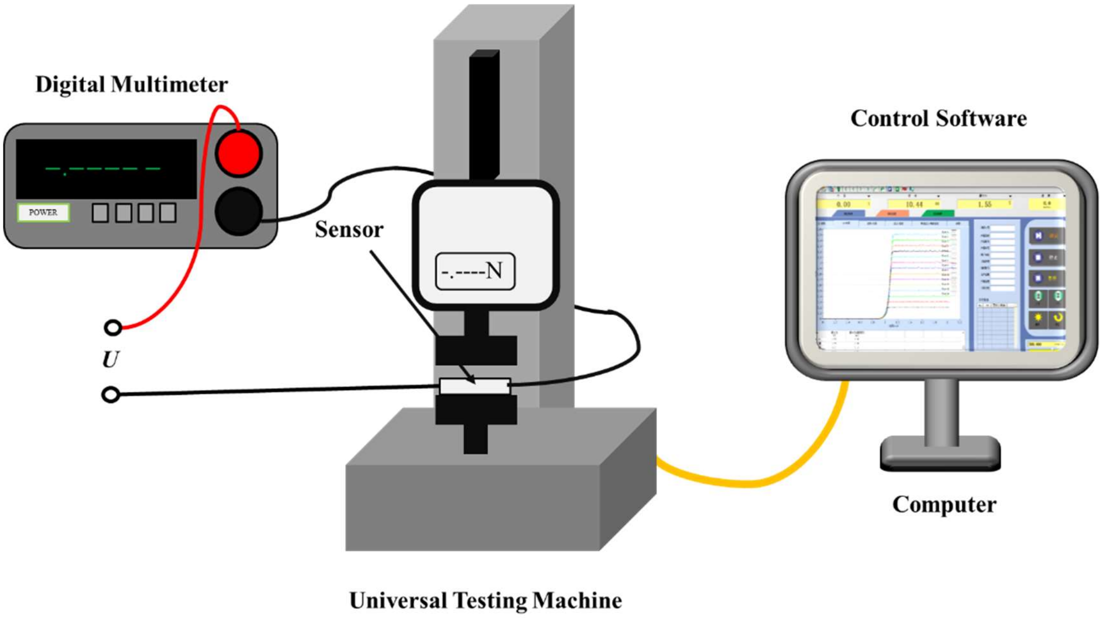

Figure S1. Schematic of the experimental device that was used for investigation of the sensing performances. In order to study the pressure sensing characteristics of the pressure sensor, a universal testing machine was used to apply the specified load on the sensor. At the same time, a computer software was employed to control the universal testing machine and record the force and corresponding output current of the sensor. The driving voltage of the measurement circuit was set to be 1 V.

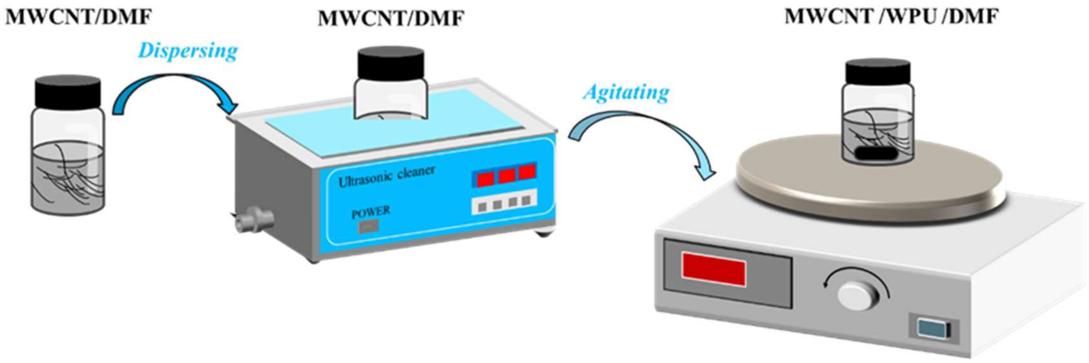

Figure S2. Preparation process of conductive MWCNTs ink.

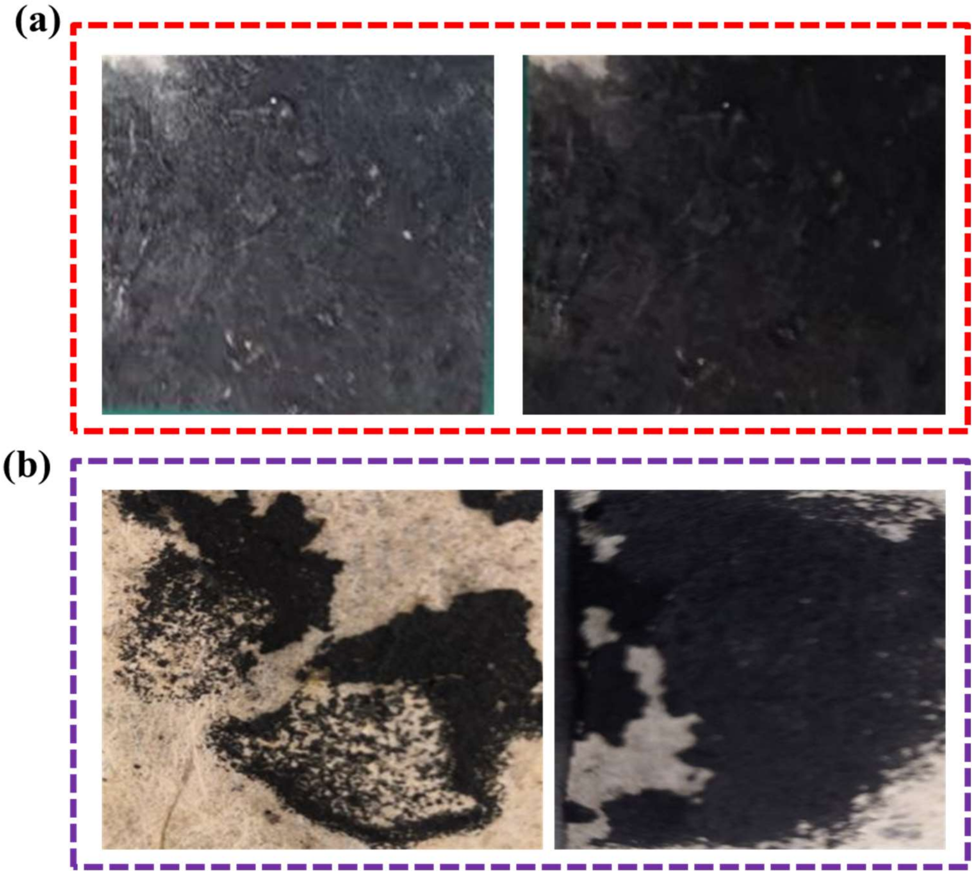

Figure S3. (a) The obtained CMP sensing layer presents highly uniform distribution of MWCNTs on surface of mulberry paper and no detachment is observed. On the contrary, the conductive MWCNTs ink without WPU shows a poor distribution, as shown in Figure (b). As can be seen from the Figure(b), MWCNTs cannot form a uniform film on mulberry paper surface and the film will shed form the mulberry paper after it dries.

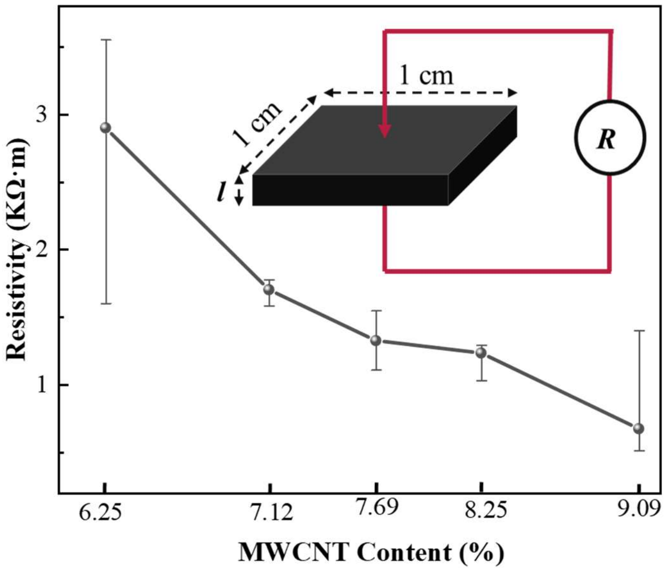

Figure S4. The resistivity of CMP Sensing layer varied with the content of MWCNTs in the conductive ink.

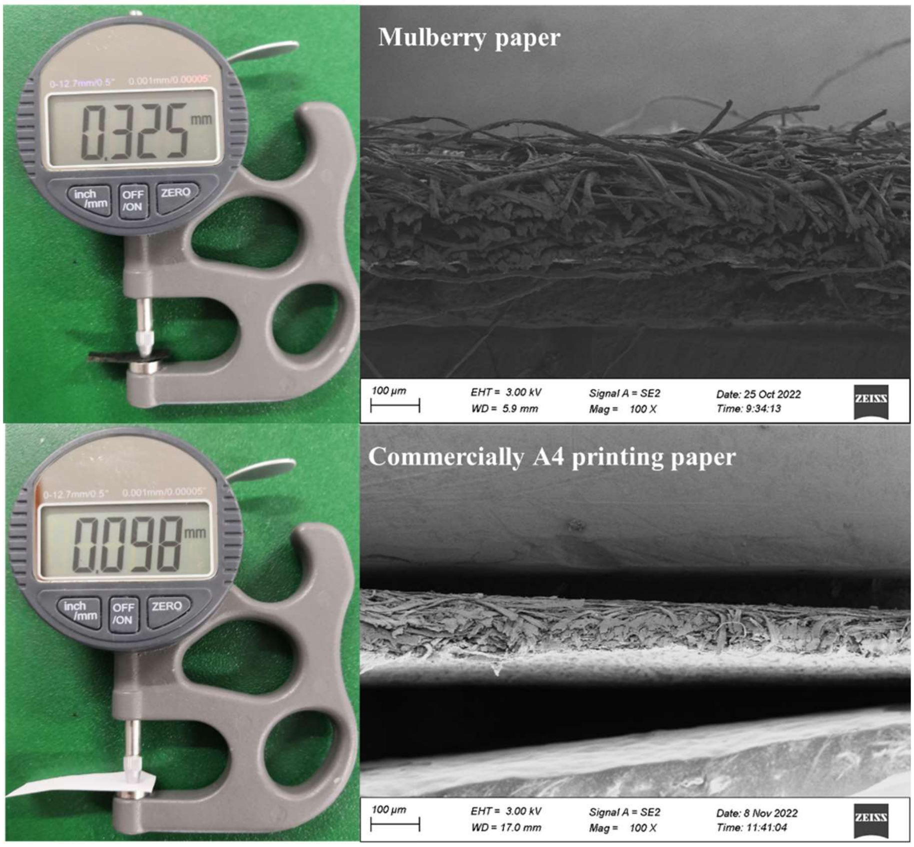

Figure S5. Cross-sectional SEM images and thickness of a piece of mulberry paper and a piece of commercial A4-printing paper.

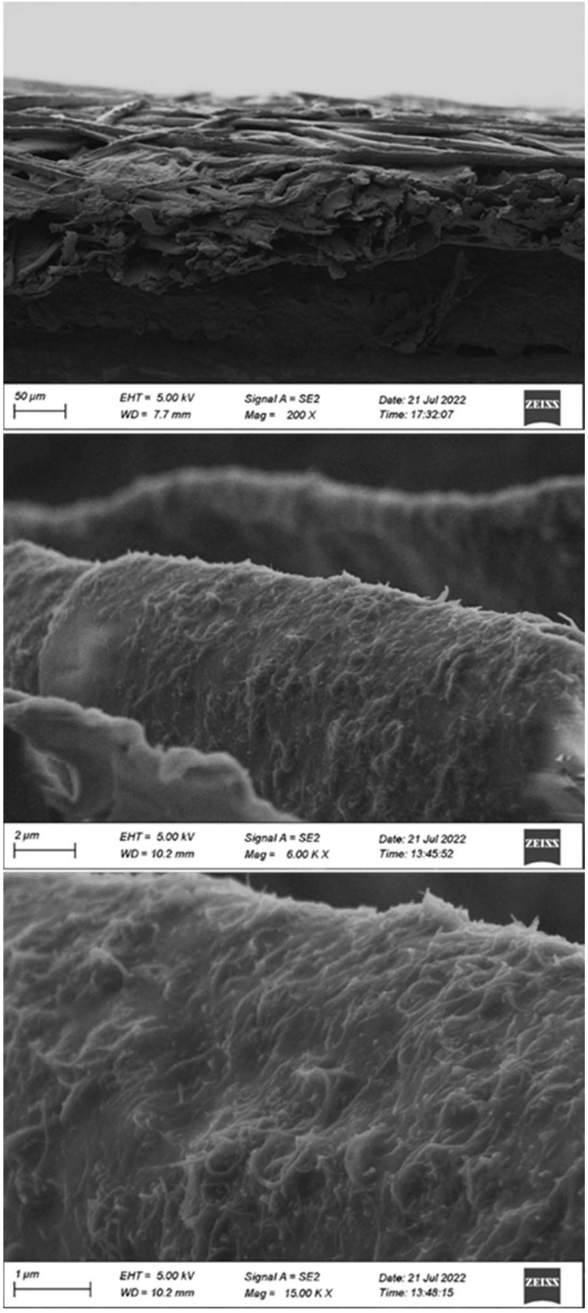

Figure S6. Cross sectional SEM images of drop coated CMP sensing layers. The images show that there are abundant carbon nanotubes distributed on the mulberry paper.

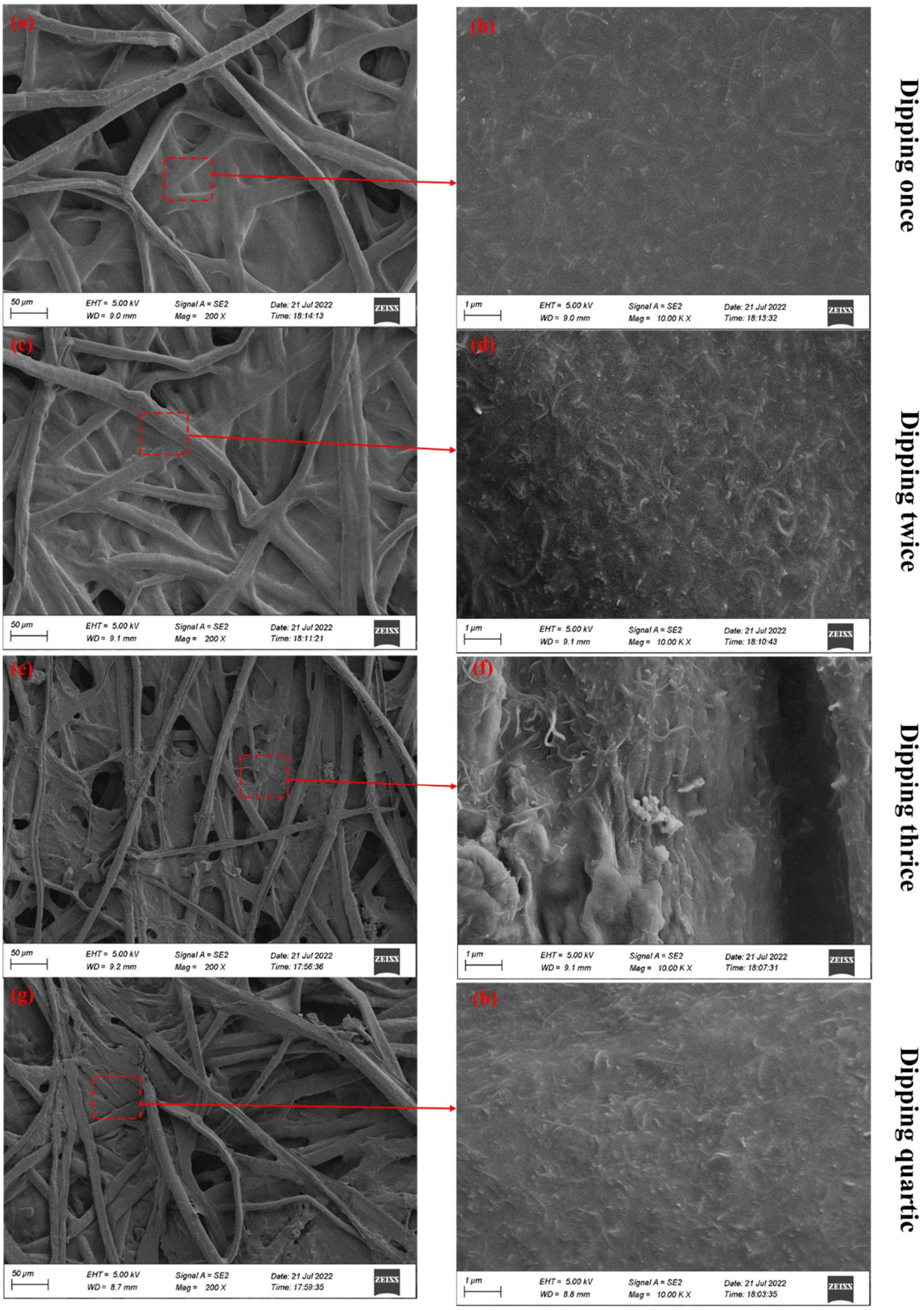

Figure S7. SEM image of CMP prepared by dip-coating process with different number of times. (a, b) Single Dip-coating. (c, d) Repeat dip-coating 2 times. (e, f) repeat dip-coating 3 times. (g, h) repeat dip-coating 4 times.

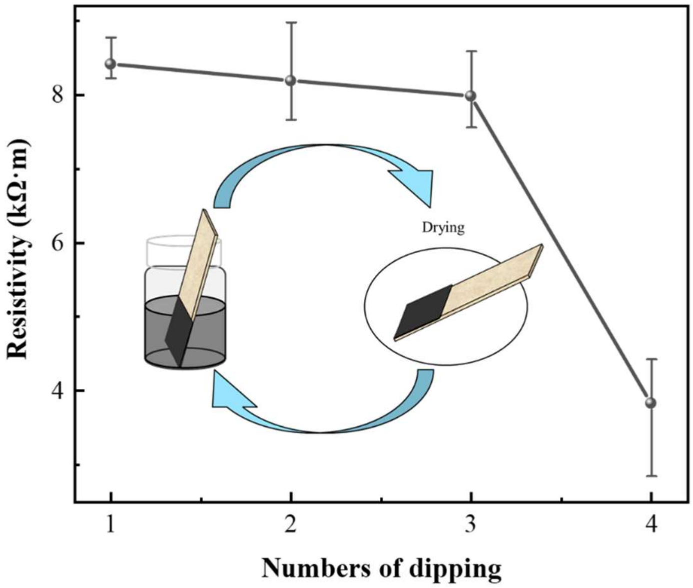

Figure S8. Resistivity of CMP changes with number of times of dip-coating. It is clearly shown that the resistivity decreases greatly after dip-coating 4 times.

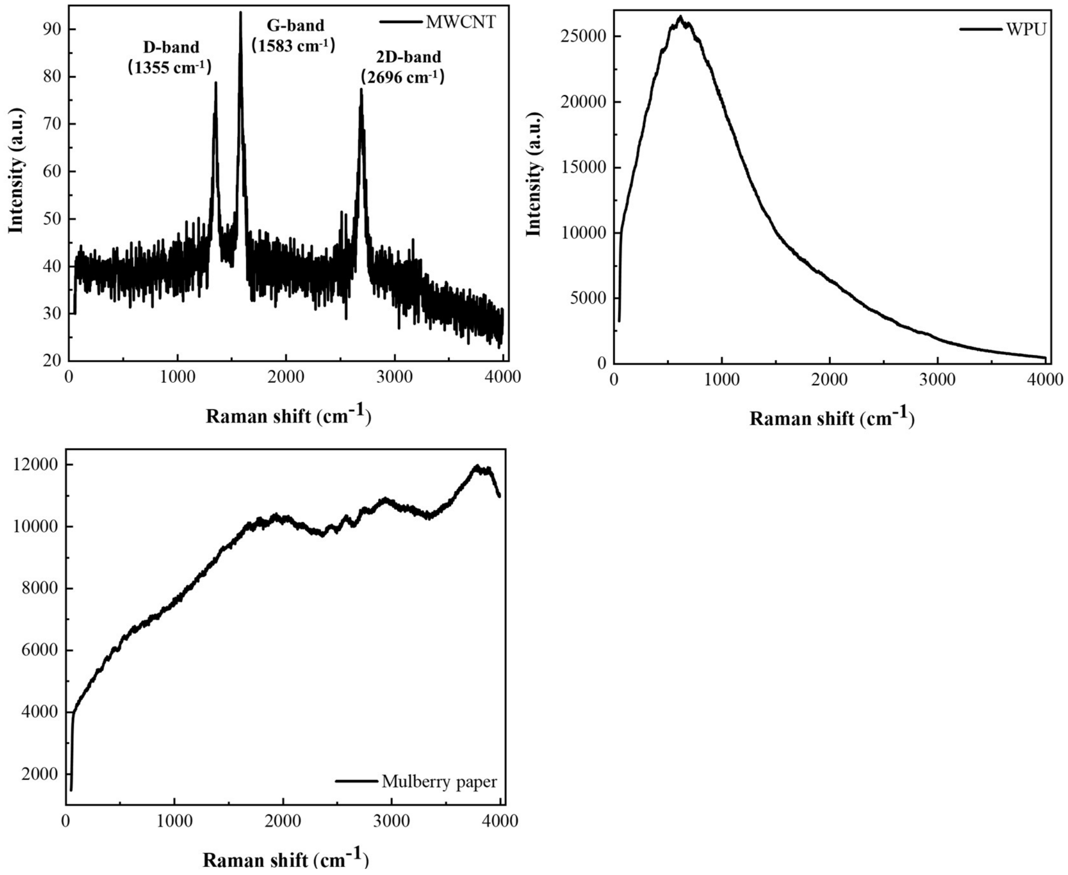

Figure S9. Raman spectra of MWCNT, WPU and pristine mulberry paper respectively. From (a), we can clearly see the Raman characteristic peaks of D - (1355 cm $^{-1}$), G - (1577 cm $^{-1}$) and 2D - (2693 cm $^{-1}$) belonging to carbon nanotubes. However, Raman spectra of WPU and mulberry paper do not show distinctly characteristic peaks.

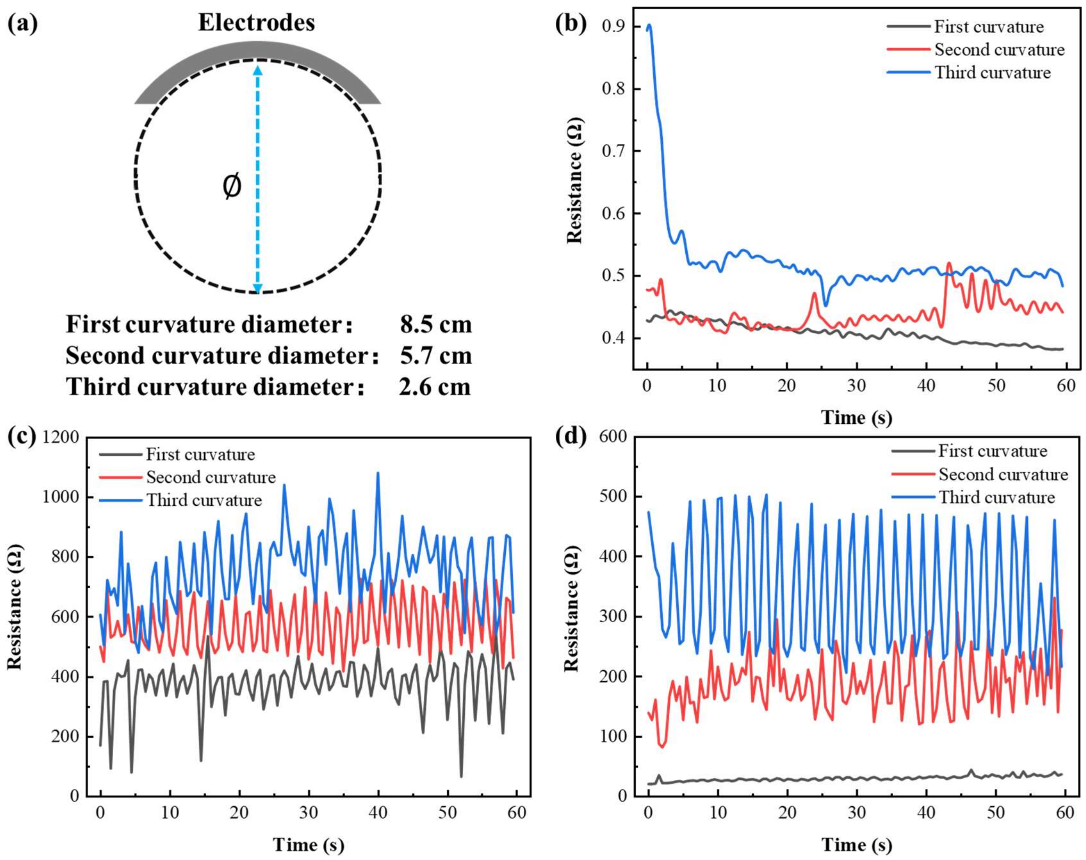

Figure S10. Reliability test for electrode on curved surfaces. (a) Schematic diagram for the test. Electrode was fixed on the surface with different curvature diameters for cycle bending test. Three curvature diameters are 8.5 cm, 5.7 cm, and 2.6 cm respectively. (b) Resistance-time curves of AgNW/FP electrodes on surfaces with three different curvatures. (c) Resistance-time curves of MWCNT/FP electrodes on surfaces with three different curvatures (MWCNTs content is 30 mg). (d) Resistance-time curves of MWCNT/FP electrodes on surfaces with three different curvatures (MWCNTs content is 40 mg).

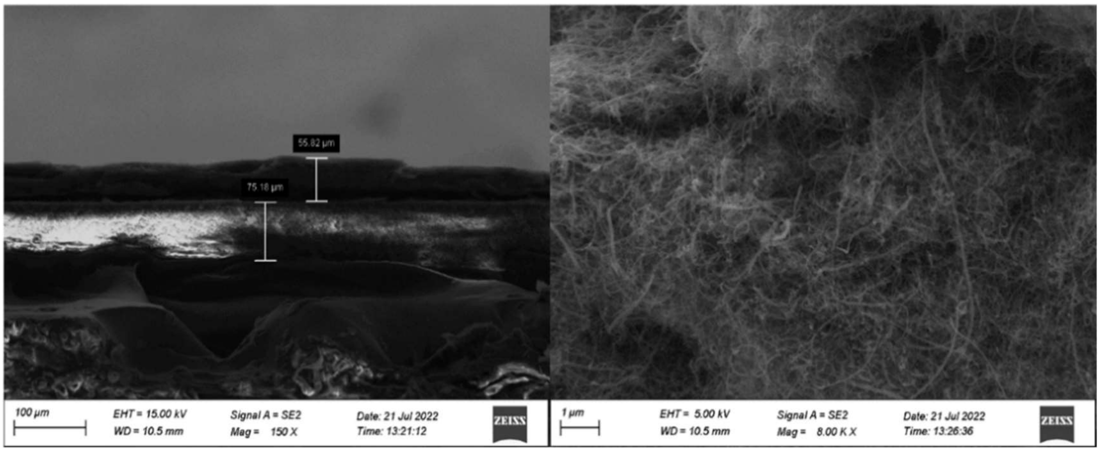

Figure S11. SEM images of MWCNT/FP electrode. The thickness of MWCNT film on the surface of a piece of filter paper is about 55.82 µm. Correspondingly, the thickness of AgNW film of the prepared AgNW/FP electrode is 47.64 µm. In addition, it is not difficult to find that MWCNTs also have an irregular distribution.

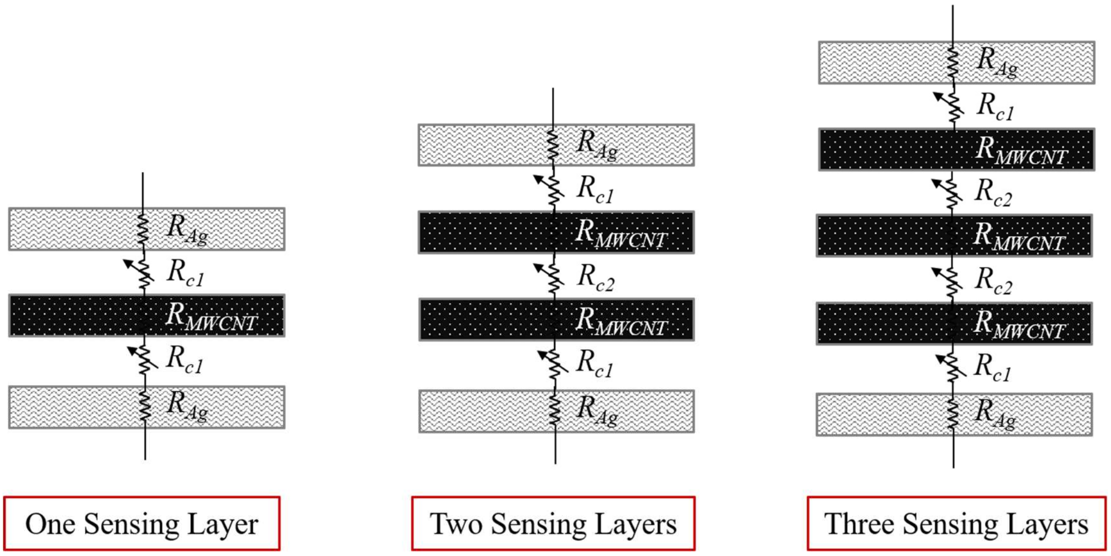

Figure S12. Schematic diagram of the equivalent circuit of sensors with one, two, and three MCMP sensing layers.

Since the sensor has a multilayer stacked structure, the total resistance of the sensor can be expressed as the sum of the resistance of the sensing material and the electrode and the contact resistance between them. As shown in Figure S12, the total resistances of the sensors with one- (R_1), two- (R_2), and three-sensing layers (R_3) can be expressed theoretically as follows:

$$R_1 = 2R_{Ag} + R_{MWCNT} + 2R_{C1} \quad (1)$$

$$R_2 = 2R_{Ag} + 2R_{MWCNT} + 2R_{C1} + R_{c2} \quad (2)$$

$$R_3 = 2R_{Ag} + 3R_{MWCNT} + 2R_{C1} + 2R_{C2} \quad (3)$$

Where R_{Ag} and R_{MWCNT} denote the resistance of AgNWs/FP electrode and MCMP sensing layer respectively. R_{C1} represents the initial contact resistance between the electrodes and sensing layer. R_{C2} denotes the contact resistance between each MCMP sensing layer. The equations (1) - (3) can be expressed according to Ohm's law as follows:

$$I_1 = V/(2R_{Ag} + R_{MWCNT} + 2R_{C1}) \quad (4)$$

$$I_2 = V/(2R_{Ag} + 2R_{MWCNT} + 2R_{C1} + R_{c2}) \quad (5)$$

$$I_3 = V/(2R_{Ag} + 3R_{MWCNT} + 2R_{C1} + 2R_{c2}\ ) \quad (6)$$

Where I_1 - I_3 is the current flowing through the corresponding sensor, and V is the driving voltage. When a pressure is applied to the sensor, the contact resistance (both the contact resistance between MCMP and AgNW/FP electrode and the contact resistance between MCMP sensing layers) is the dominant factor of the total resistance change. Therefore, the multilayer stacked MCMP can be considered as a variable resistor, and the current of the sensor under pressure can be expressed as:

$$I_1^* = V/(2R_{Ag} + R_{MWCNT} + 2R_{C1}^*) \quad (7)$$

$$I_2^* = V/(2R_{Ag} + 2R_{MWCNT} + 2R_{C1}^* + R_{C2}^*) \quad (8)$$

$$I_3^* = V/(2R_{Ag} + 3R_{MWCNT} + 2R_{C1}^* + 2R_{C2}^*\ ) \quad (9)$$

Where I_1^*, I_2^*, and I_3^* represent the current flowing through the sensor under a loading pressure. R_{C1}^* and R_{C2}^* represent the contact resistance between AgNW/FP electrode and MCMP and the contact resistance between MCMP sensing layers, respectively.

The current change ($\Delta I = |\ I_1^*\ -\ I_1\ |$) of a one-layered sensor is expressed as follows :

$$\Delta I_1 = \frac{V}{2R_{Ag} + R_{MWCNT} + 2R_{CI}^*} - \frac{V}{2R_{Ag} + R_{MWCNT} + 2R_{C1}}$$
$$= \frac{2V(R_{C1} - R_{C1}^*)}{[(2R_{Ag} + R_{MWCNT} + 2R_{CI}^*)(2R_{Ag} + R_{MWCNT} + 2R_{C1})]} \quad (10)$$

Based on the definition of piezoresistive sensitivity [$S = (\Delta I/I)/P$], the sensitivity (S_1) of a one-layer sensor can be expressed as :

$$S_1 = \frac{2(R_{C1} - R_{C1}^*)}{\left(2R_{Ag} + R_{MWCNT} + 2R_{CI}^*\right) * P} \quad (11)$$

Where P is the applied pressure.

Similarly, the sensitivity of the sensor with two-(S_2) and three-(S_3) sensing layers can be expressed as follows:

$$S_2 = \frac{2(R_{C1} - R_{C1}^*) + (R_{C2} - R_{C2}^*)}{\left(2R_{Ag} + 2R_{MWCNT} + 2R_{CI}^* + R_{C2}^*\right) * P} \quad (12)$$

$$S_3 = \frac{2(R_{C1} - R_{C1}^*) + 2(R_{C2} - R_{C2}^*)}{\left(2R_{Ag} + 3R_{MWCNT} + 2R_{CI}^* + 2R_{C2}^*\right) * P} \quad (13)$$

According to equations (11) – (13), the sensitivity of the sensor depends on multiple parameters, including the resistance of the MCMP sensing layer, the contact resistance between MCMP layers, as well as the contact resistance between MCMP layers and the electrodes. The change of contact area caused by the increase of layers can directly lead to a significant increase in contact resistance. Consequently, the sensitivity of the sensor increases along with the increasing number of MCMP layers. This shows that the actual sensitivities of sensors with one-, two-, and three-sensing layers may be higher than the theoretical values after model simplification.

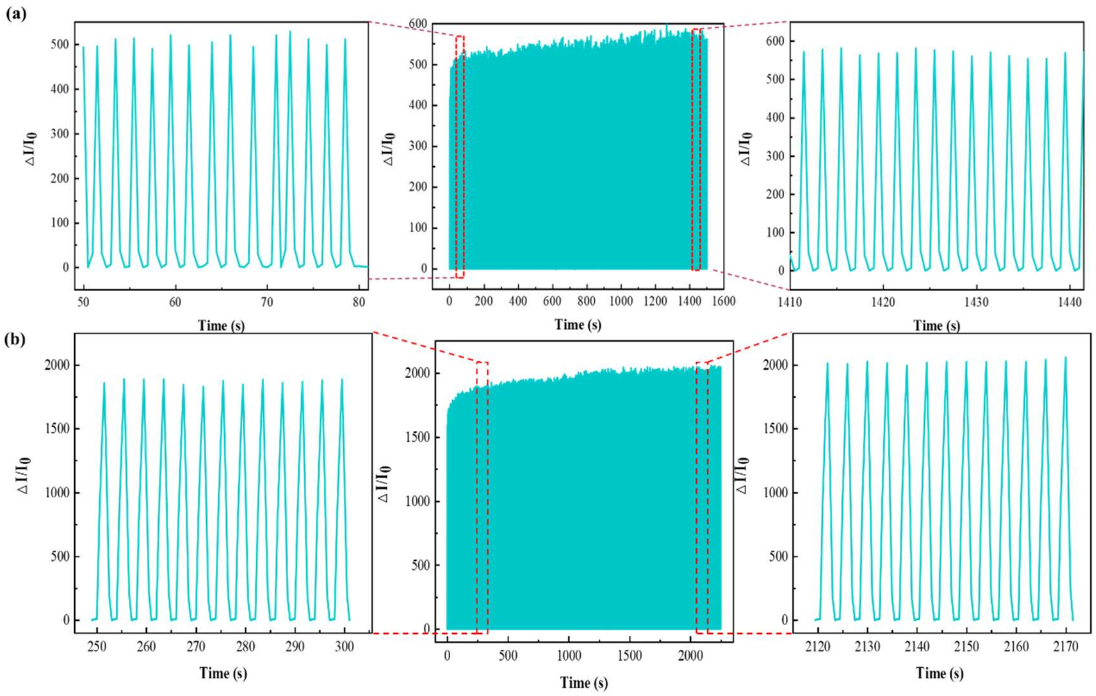

Figure S13. Repeatability test of the presented pressure sensor for repeated loading of 1000 cycles. (a) Loading pressure is 100 kPa; (b) Loading pressure is 500 kPa. It is obvious that the relative current change is almost constant, which indicates that the sensor has good stability and reliability.

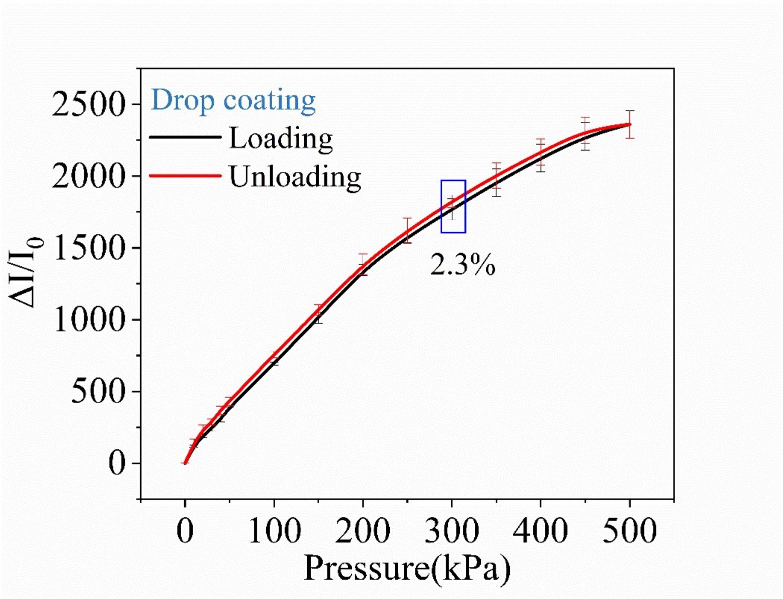

(a)

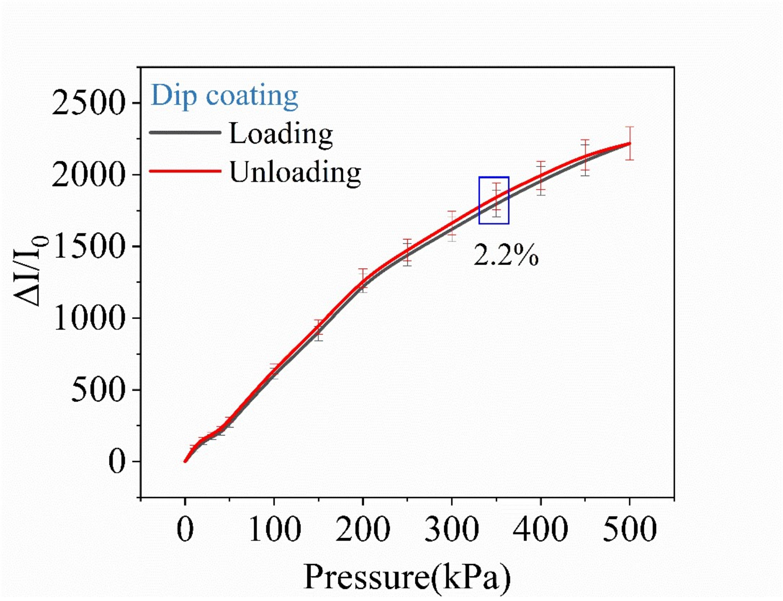

(b)

Figure S14. Hysteresis test of the presented sensor by repeating the loading and unloading process for 6 times. The maximum hysteresis error of the sensor prepared using the drop coating method and the dip coating method are calculated to be about 2.3% and 2.2% respectively.

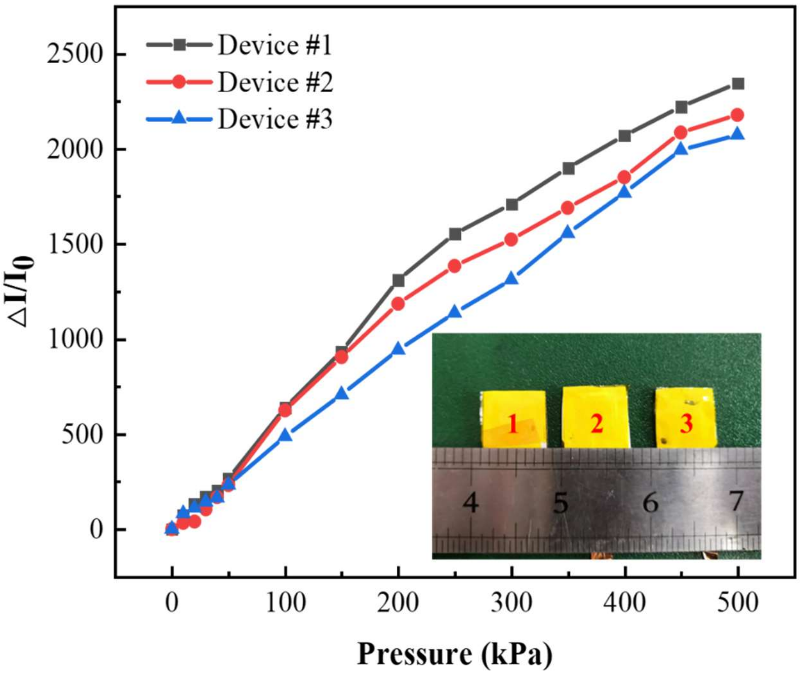

Figure S15. Pressure sensing performance of three different sensors prepared by the same method. Although sensors present a slightly different pressure sensing performances, all devices characterize high sensitivity in a wide sensing range. This result demonstrates the significant advantages of our all paper based pressure sensors.

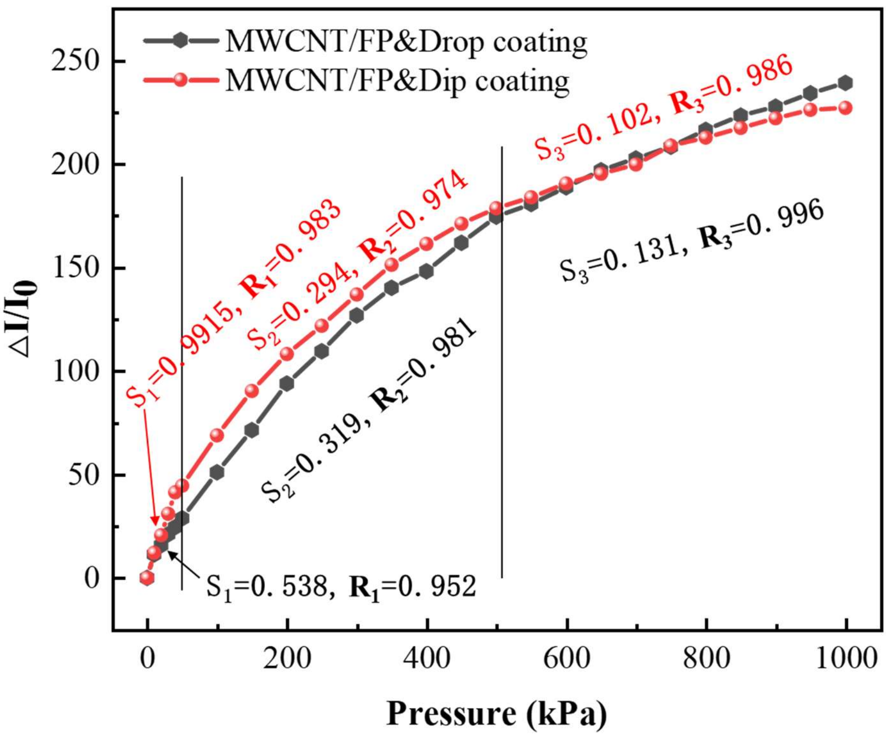

Figure S16. Relative change in current of the three layers CMP sensor with MWCNT/FP electrodes. CMP sensing layers were prepared by dip coating and drop coating respectively, and packaged with MWCNT/FP electrodes to realize corresponding sensor.

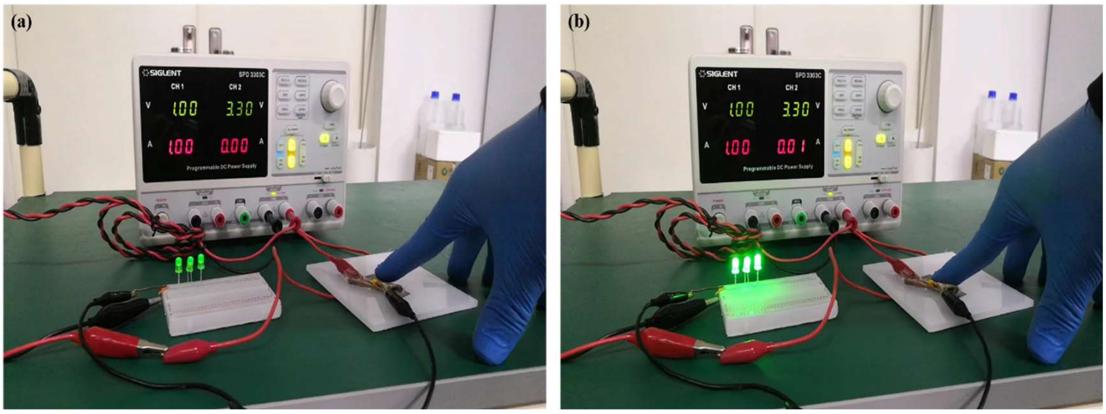

Figure S17. A designed sensor is used to control the brightness of the LEDs. (a) The current in the circuit is very small and the brightness of LEDs is very low when the sensor is not subjected to a pressure. (b) As the sensor is pressed, the current in the circuit increases and LEDs are getting brighter accordingly.

Table S1. Composition of sensing layers with different content of MWCNTs.

Sample name	MWCNT(g)	WPU(g)	DMF(mL)
$MWCNT_{6.25\%}$/WPU	0.020	0.3	3
$MWCNT_{7.12\%}$/WPU	0.023	0.3	3
$MWCNT_{7.69\%}$/WPU	0.025	0.3	3
$MWCNT_{8.25\%}$/WPU	0.027	0.3	3
$MWCNT_{9.09\%}$/WPU	0.030	0.3	3

Table S2. Comparison of pressure-sensing performances with respect to sensitivity and sensing range among reported paper-based tactile sensors.

Sensing Material	Operation type	Sensitivity (kPa^{-1})	Linear sensing range (kPa)	Ref.
MWCNT/WPU, AgNWs Mulberry paper , Filter paper	*Resistive*	*6.26, 4.29,and 1.707*	*0-50,50-500,and 500-1000*	*This work*
**AgNWs/PDMS/paper**	**Capacitive**	**1.05 and 0.015**	**0-2 and 2-15**	**[54]**
**Pyrrole/PDMS/tissue paper**	**Capacitive**	**0.96 and 0.09**	**0-1.76 and 1.76-22**	**[55]**
**Graphite/ tissue paper**	**Capacitive**	**0.63 and 0.14**	**0-2 and 2-50**	**[56]**
**AuNWs/PDMS/tissue paper**	**Resistive**	**1.14**	**0-5**	**[57]**
**Single-wall Carbon Nanotube /tissue paper**	**Resistive**	**2.2 and 1.3**	**0.035-2.5 and 2.5-11.7**	**[58]**
**WSe_2 nanosheets/tissue paper**	**Resistive**	**1911.4 and 9.8**	**0–0.75 and 0.75-15**	**[59]**
**Graphene oxide(GO) / tissue paper**	**Resistive**	**17.2 and 0.012**	**0-2 and 2-20**	**[60]**
**Silver paste/ crepe paper (tissue paper)**	**Resistive**	**5.67， 2.52， 0.87, and 0.32**	**0–0.42, 0.42–2.53, 2.53–8.75 and 8.75–20**	**[61]**
**Mxene/ Polylactic Acid (PLA)/tissue paper**	**Resistive**	**0.55, 3.81, and 2.52**	**0.023-0.982, 0.982-10, and 10-30**	**[15]**
**silver nanowires(AgNW)/nanocellulose paper**	**Resistive**	**1.5**	**0.03–30.2**	**[62]**
**PPy/tissue papers**	**Resistive**	**4.8 and 1.7**	**0-5.5 and 5.5-40**	**[63]**
**Carbon ink/A4 printing paper**	**Resistive**	**0.614 and 0.064**	**0-6 and 6-40**	**[64]**
**3,4-ethylenedioxythiophene (EDOT) /$AgNO_3$ / filter paper**	**Resistive**	**0.119 and 0.031**	**0-12 and 12-40**	**[65]**
**WSe2/ Whatman filter paper**	**Resistive**	**29.24**	**1–100**	**[66]**
**Mxene/AgNWs/ tissue paper**	**Resistive**	**509.5, 179.4 , and 53.7**	**0.5-10, 10-30, and 30-100**	**[41]**
**PEDOT:PSS/tissue paper**	**Resistive**	**1.14 and 0.6**	**0-100 and 100-300**	**[67]**
